# Substance P Promotes Leukocyte Infiltration in the Liver and Lungs of Mice with Sepsis: A Key Role for Adhesion Molecules on Vascular Endothelial Cells

**DOI:** 10.3390/ijms25126500

**Published:** 2024-06-13

**Authors:** Zhixing Zhu, Stephen Chambers, Madhav Bhatia

**Affiliations:** Department of Pathology and Biomedical Science, University of Otago, Christchurch 8140, New Zealand; zhixing.zhu@postgrad.otago.ac.nz (Z.Z.); steve.chambers@otago.ac.nz (S.C.)

**Keywords:** adhesion molecules, leukocyte infiltration, neurokinin-1 receptor, substance P, vascular endothelial cells

## Abstract

Substance P (SP), encoded by the *Tac1* gene, has been shown to promote leukocyte infiltration and organ impairment in mice with sepsis. Neurokinin-1 receptor (NK1R) is the major receptor that mediates the detrimental impact of SP on sepsis. This investigation studied whether SP affects the expression of adhesion molecules, including intercellular cell adhesion molecule-1 (ICAM1) and vascular cell adhesion molecule-1 (VCAM1) on vascular endothelial cells in the liver and lungs, contributing to leukocyte infiltration in these tissues of mice with sepsis. Sepsis was induced by caecal ligation and puncture (CLP) surgery in mice. The actions of SP were inhibited by deleting the *Tac1* gene, blocking NK1R, or combining these two methods. The activity of myeloperoxidase and the concentrations of ICAM1 and VCAM1 in the liver and lungs, as well as the expression of ICAM1 and VCAM1 on vascular endothelial cells in these tissues, were measured. The activity of myeloperoxidase and the concentration of ICAM1 and VCAM1 in the liver and lungs, as well as the expression of ICAM1 and VCAM1 on vascular endothelial cells in these tissues, increased in mice with CLP surgery-induced sepsis. Suppressing the biosynthesis of SP and its interactions with NK1R attenuated CLP surgery-induced alterations in the liver and lungs of mice. Our findings indicate that SP upregulates the expression of ICAM1 and VCAM1 on vascular endothelial cells in the liver and lungs, thereby increasing leukocyte infiltration in these tissues of mice with CLP surgery-induced sepsis by activating NK1R.

## 1. Introduction

A dysregulated inflammatory response in the host is a significant contributor to the pathogenesis of sepsis and associated organ dysfunction [1,2,3]. The mechanism leading to the dysregulated inflammatory response during sepsis remains poorly understood [4]. Substance P (SP), encoded by the *tachykinin 1* (*Tac1*) gene in rodents [5], has been demonstrated to be an important mediator contributing to the dysregulation of the acute inflammatory response in the liver and lungs of mice with sepsis [6]. The pro-inflammatory actions of SP in sepsis-associated acute liver and lung injury are mainly mediated by neurokinin-1 receptor (NK1R) [6]. However, the precise mechanism leading to the detrimental effects of SP on sepsis remains to be investigated.

Recently, we have shown that increased SP-NK1R signalling contributes to the upregulation of leukocyte infiltration and acute inflammatory injury in the liver and lungs of mice with sepsis induced by caecal ligation and puncture (CLP) surgery [6]. The recruitment of leukocytes to the sites of injury from the bloodstream is a complex process that involves many (patho)physiological mechanisms [7,8,9,10,11]. The role of vascular endothelial cells in the initiation, progression, and resolution of an inflammatory response in the host has been known for a long time [7,12,13,14,15]. Upon activation by pro-inflammatory cytokines, primarily tumour necrosis factor (TNF)-α and interleukin (IL)-1β, vascular endothelial cells express higher concentrations of adhesion molecules, including intercellular adhesion molecule 1 (ICAM1) and vascular adhesion molecule 1 (VCAM1) [7]. The increased expression of ICAM1 and VCAM1 enhances the interactions between vascular endothelial cells and circulating leukocytes, thereby promoting the infiltration of leukocytes and subsequently contributing to a dysregulated inflammatory response in the host [7]. Accumulating evidence suggests that vascular endothelial dysfunction is a critical contributor to the pathogenesis of an acute inflammatory response and organ dysfunction in sepsis [16,17,18,19]. Nonetheless, it is unclear whether the abnormality in vascular endothelial cells also contributes to the SP-mediated upregulation of leukocyte infiltration in the liver and lungs of mice with sepsis induced by CLP surgery.

Liver sinusoidal endothelial cells are highly specialized liver vascular endothelial cells [20,21]. Abnormalities of the liver sinusoidal endothelial cells, including defenestration and increased gap formation, have been linked to the pathogenesis of acute liver injury in mice with CLP surgery-induced sepsis [22,23]. In contrast, protecting these vascular endothelial cells attenuated CLP surgery-induced acute liver injury in mice [22,23]. Dysfunction of pulmonary vascular endothelial cells has also been recognized as a crucial mechanism of sepsis-associated acute lung injury [23,24,25]. The deletion of the *Tac1* gene has been shown to protect mice from sepsis-related liver sinusoidal endothelial cell damage, linking the abnormal biosynthesis of SP to the dysfunction of vascular endothelial cells in the liver in sepsis [26]. The increased SP-NK1R signalling has been implicated in the upregulated production of cytokines, including TNF-α and IL-1β, in the liver and lungs of mice with sepsis induced by CLP surgery [6]. These studies collectively identified the possible involvement of SP in the activation of vascular endothelial cells in these tissues of mice with CLP surgery-induced sepsis by activating NK1R. Recently, CLP surgery was shown to upregulate the expression of ICAM1 and VCAM1 on liver sinusoidal endothelial cells and pulmonary vascular endothelial cells in mice [27]. Suppressing the biosynthesis of hydrogen sulfide attenuated the CLP surgery-induced increase in the expression of ICAM1 and VCAM1 on vascular endothelial cells in the liver and lungs, resulting in reduced leukocyte infiltration in these tissues of mice [27]. The SP-NK1R cascade has been demonstrated to be a significant downstream mechanism underlying the pro-inflammatory actions of endogenous hydrogen sulfide in the liver and lungs of mice with CLP surgery-induced sepsis [28]. However, uncertainties remain regarding whether SP activates liver sinusoidal endothelial cells and pulmonary vascular endothelial cells to express higher concentrations of ICAM1 and VCAM1, thereby promoting leukocyte infiltration in the liver and lungs of mice with sepsis induced by CLP surgery.

This study aimed to determine whether SP upregulates the expression of ICAM1 and VCAM1 on vascular endothelial cells in the liver and lungs, contributing to increased infiltration of leukocytes in these tissues of mice with sepsis induced by CLP surgery. This investigation included determining whether NK1R is involved in the actions played by SP in activating vascular endothelial cells in the liver and lungs of mice with sepsis induced by CLP surgery. To achieve these aims, three complimentary approaches—the genetic deletion of the *Tac1* gene, the pharmacological blockade of NK1R using L703606, and the combination of these two approaches—were used to suppress the actions of SP in mice. The activity of myeloperoxidase (MPO) and the concentration of ICAM1 and VCAM1 in the liver and lungs, as well as the expression of ICAM1 and VCAM1 on vascular endothelial cells in these tissues, were measured.

## 2. Results

### 2.1. Inhibiting the Biosynthesis of SP or Blocking NK1R Mitigated the Elevation in the Activity of MPO in the Liver of Mice with Sepsis Induced by CLP Surgery

**Figure 1:**
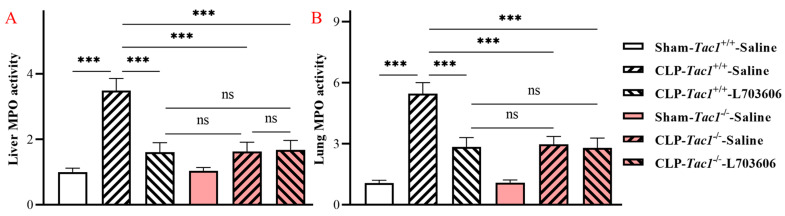
The activity of MPO in the liver and lungs of mice following CLP surgery-induced sepsis. The activity of MPO was expressed as the fold increase over the sham-operated control. The activity of MPO in the liver (**A**) and lungs (**B**) of the *Tac1*^+/+^ mice was significantly upregulated by CLP surgery. This increase was attenuated by the suppression of SP biosynthesis or NK1R blockade. One-way ANOVA with Tukey’s multiple comparisons test were applied to evaluate the significance of differences among different groups. *** *p* < 0.001, ns: not significant. Data are presented as means ± SDs (*n* = 6).

As depicted in Figure 1A, the activity of MPO in the liver of mice with sepsis induced by CLP surgery was significantly higher than in the sham-operated controls (3.48-fold increase, *p* < 0.001). Inhibiting the biosynthesis of SP by the deletion of the *Tac1* gene, (2.15-fold decrease, *p* < 0.001), blocking the action of NK1R (using L703606, 2.17-fold decrease, *p* < 0.001), or combining these two methods (2.08-fold decrease, *p* < 0.001) significantly attenuated the CLP surgery-induced elevation in the activity of MPO in the mouse liver.

### 2.2. Inhibiting the Biosynthesis of SP or Blocking NK1R Attenuated the Increase in the Activity of MPO in the Lungs of Mice with Sepsis Induced by CLP Surgery

CLP surgery significantly increased the activity of MPO in the lungs of mice when compared with sham-operated mice (5.13-fold increase, *p* < 0.001), as illustrated in Figure 1B. The suppression of SP biosynthesis by the deletion of the *Tac1* gene, (2.15-fold decrease, *p* < 0.001), blocking NK1R (using L703606, 2.17-fold decrease, *p* < 0.001), or combining these two methods (2.08-fold decrease, *p* < 0.001) significantly attenuated the CLP surgery-induced elevation in the activity of MPO in the lungs of mice.

### 2.3. Inhibiting the Biosynthesis of SP or Blocking NK1R Attenuated the Increased Concentrations of ICAM1 and VCAM1 in the Liver of Mice with Sepsis Induced by CLP Surgery

**Figure 2:**
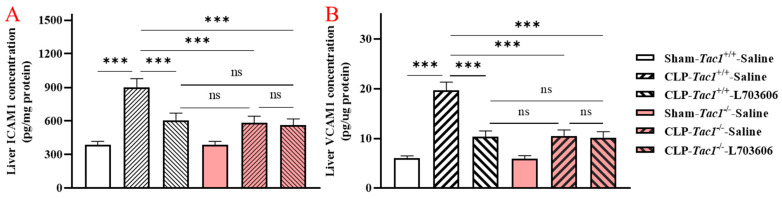
The concentrations of ICAM1 and VCAM1 in the liver of mice with CLP surgery-induced sepsis. The expression of ICAM1 (**A**) and VCAM1 (**B**) in the liver of the *Tac1*^+/+^ mice was significantly upregulated by the CLP surgery. This increase was attenuated by the suppression of SP biosynthesis or NK1R blockade. One-way ANOVA with Tukey’s multiple comparisons test were applied to evaluate the significance of differences among different groups. *** *p* < 0.001, ns: not significant. Data are presented as means ± SDs (*n* = 6).

As illustrated in Figure 2, the concentration of ICAM1 in the liver of mice with sepsis induced by CLP surgery was significantly higher (*p* < 0.001) than in the sham-operated controls. The elevation in the concentration of ICAM1 in the liver of mice with sepsis induced by the CLP surgery was reduced by inhibiting the biosynthesis of SP through the deletion of the *Tac1* gene, (*p* < 0.001), blocking the actions played by NK1R (using L703606, *p* < 0.001), or combining these two methods (*p* < 0.001). Likewise, the CLP surgery significantly increased the concentration of VCAM1 in the liver of mice (*p* < 0.001) compared with the sham-operated controls. Suppressing SP biosynthesis via deleting the *Tac1* gene (*p* < 0.001), blocking NK1R using L703606 (*p* < 0.001) or combining these two methods (*p* < 0.001) also reduced the concentration of VCAM1 in the liver of mice with sepsis induced by CLP surgery compared to the controls.

### 2.4. Inhibiting the Biosynthesis of SP or Blocking NK1R Attenuated the Increases in the Concentrations of ICAM1 and VCAM1 in the Lungs of Mice with Sepsis Induced by CLP Surgery

**Figure 3:**
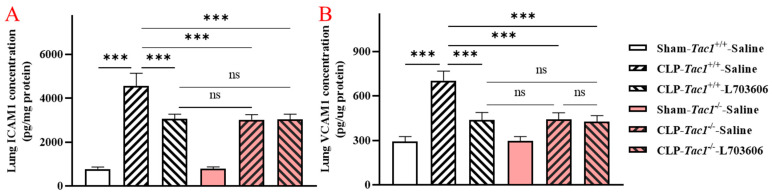
The concentrations of ICAM1 and VCAM1 in the lungs of mice with CLP surgery-induced sepsis. The expression of ICAM1 (**A**) and VCAM1 (**B**) in the lungs of the *Tac1*^+/+^ mice was significantly upregulated by the CLP surgery. This increase was attenuated by the suppression of SP biosynthesis or NK1R blockade. One-way ANOVA with Tukey’s multiple comparisons test were applied to evaluate the significance of differences among different groups. *** *p* < 0.001, ns: not significant. Data are presented as means ± SDs (*n* = 6).

As depicted in Figure 3, the concentration of ICAM1 in the lungs of mice with sepsis induced by CLP surgery was significantly higher (*p* < 0.001) compared with the concentrations found in sham-operated controls The elevation in the concentration of ICAM1 in the lungs of mice with sepsis induced by the CLP surgery was attenuated by inhibiting the biosynthesis of SP through the deletion of the *Tac1* gene, (*p* < 0.001), blocking the action of NK1R (using L703606, *p* < 0.001), or combining these two methods (*p* < 0.001). Likewise, compared with the sham operation, the CLP surgery significantly increased the concentration of VCAM1 in the lungs of mice (*p* < 0.001). Suppressing SP biosynthesis via deleting the *Tac1* gene (*p* < 0.001), blocking NK1R using L703606 (*p* < 0.001) or combining these two methods (*p* < 0.001) also attenuated the increase in the concentration of VCAM1 in the lungs of mice with sepsis induced by CLP surgery (*p* < 0.001).

### 2.5. Inhibiting the Biosynthesis of SP or Blocking NK1R Attenuated the Upregulation of the Expression of ICAM1 and VCAM1 on the Liver Sinusoidal Endothelial Cells of Mice with Sepsis Induced by CLP Surgery

**Figure 4:**
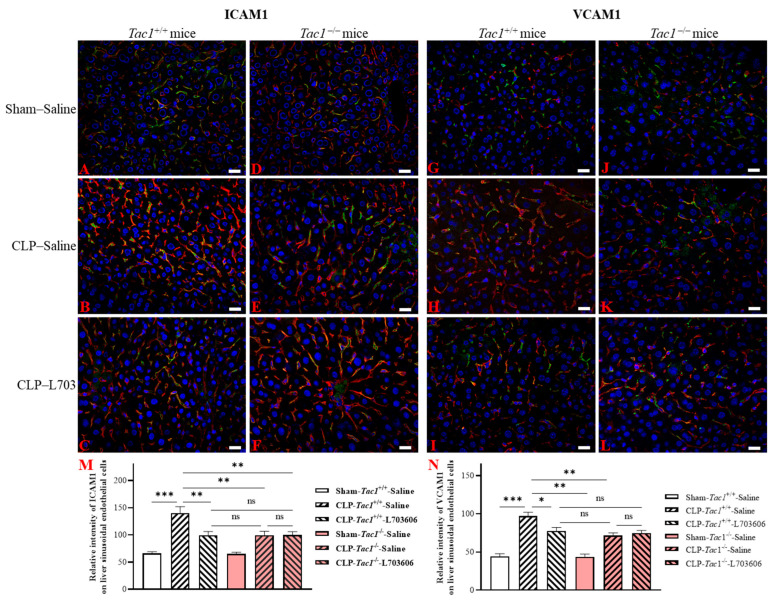
The expression of ICAM1 (red) and VCAM1 (red) on liver sinusoidal endothelial cells (green) of mice. Representative images of ICAM1 and VCAM1 from group 1 to group 6 are shown in panels (**A**–**F**) and panels (**G**–**L**), respectively. Scale bars represent 20 μm. The change in the expression of ICAM1 and VCAM1 on the liver sinusoidal endothelial cells was quantitated and presented in panels (**M** and **N**), respectively. Compared with the sham operation, CLP surgery upregulated the expression of ICAM1 and VCAM1 on the liver sinusoidal endothelial cells of mice. This increase was attenuated by *Tac1* gene deletion, NK1R blockade, and combining these two methods. One-way ANOVA with Tukey’s multiple comparisons test were applied to evaluate the significance of differences among different groups. * *p* < 0.05, ** *p* < 0.01, *** *p* < 0.001, and ns: not significant. Data are presented as means ± SEMs (*n* = 6).

As illustrated in Figure 4, the expression of ICAM1 on the liver sinusoidal endothelial cells was significantly elevated in the *Tac1*^+/+^ mice with sepsis induced by CLP surgery compared with the sham-operated controls (2.13-fold increase, *p* < 0.001). In contrast, the suppression of SP biosynthesis by the deletion of the *Tac1* gene, (1.42-fold decrease, *p* < 0.01), the pharmacological blockade of NK1R (using L703606, 1.42-fold decrease, *p* < 0.01), or the combination of these two methods (1.40-fold decrease, *p* < 0.01) attenuated the CLP surgery-induced upregulation of the expression of ICAM1 on the vascular endothelial cells in the liver of mice. The expression of VCAM1 on the liver sinusoidal endothelial cells was also significantly upregulated in the *Tac1*^+/+^ mice with sepsis induced by CLP surgery compared with the sham-operated controls (2.20-fold increase, *p* < 0.001), as depicted in Figure 4. In contrast, the suppression of SP biosynthesis by the deletion of the *Tac1* gene, (1.35-fold decrease, *p* < 0.01), the pharmacological blockade of NK1R (using L703606, 1.25-fold decrease, *p* < 0.05), or the combination of these two methods (1.30-fold decrease, *p* < 0.01) attenuated the CLP surgery-induced upregulation of the expression of VCAM1 on the vascular endothelial cells in the liver of mice.

### 2.6. Inhibiting the Biosynthesis of SP or Blocking NK1R Attenuated the Upregulation of the Expression of ICAM1 and VCAM1 on the Pulmonary Endothelial Cells of Mice with Sepsis Induced by CLP Surgery

**Figure 5:**
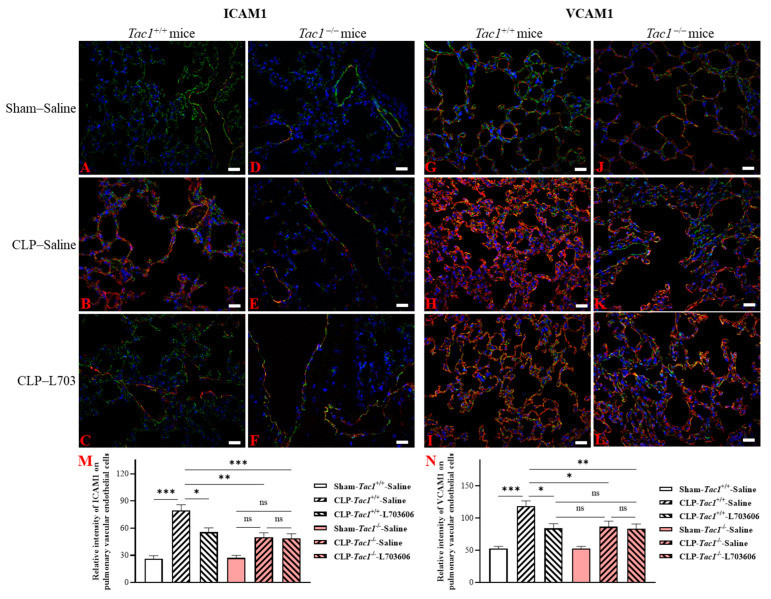
The expression of ICAM1 (red) and VCAM1 (red) on the pulmonary vascular endothelial cells (green) of mice. Representative images of ICAM1 and VCAM1 from group 1 to group 6 are shown in panels (**A**–**F**) and panels (**G**–**L**), respectively. Scale bars represent 20 μm. The change in the expression of ICAM1 and VCAM1 on the pulmonary vascular endothelial cells was quantitated and presented in panels (**M** and **N**), respectively. Compared with the sham operation, CLP surgery upregulated the expression of ICAM1 and VCAM1 on the pulmonary vascular endothelial cells of mice. This increase was attenuated by *Tac1* gene deletion, NK1R blockade, and combining these two methods. One-way ANOVA with Tukey’s multiple comparisons test were applied to evaluate the significance of differences among different groups. * *p* < 0.05, ** *p* < 0.01, *** *p* < 0.001, and ns: not significant. Data are presented as means ± SEMs (*n* = 6).

The expression of ICAM1 on pulmonary vascular endothelial cells was significantly increased in the *Tac1*^+/+^ mice with sepsis induced by CLP surgery compared with the sham-operated controls (2.13-fold increase, *p* < 0.001), as depicted in Figure 5. In contrast, the suppression of SP biosynthesis by the deletion of the *Tac1* gene, (1.42-fold decrease, *p* < 0.01), the pharmacological blockade of NK1R (using L703606, 1.42-fold decrease, *p* < 0.01), or the combination of these two methods (1.40-fold decrease, *p* < 0.01) attenuated the CLP surgery-induced upregulation of the expression of ICAM1 on the vascular endothelial cells in the lungs of mice. As shown in Figure 5, the expression of VCAM1 on the pulmonary vascular endothelial cells was also significantly upregulated in the *Tac1*^+/+^ mice with sepsis induced by CLP surgery compared with the sham-operated controls (2.20-fold increase, *p* < 0.001). In contrast, the suppression of SP biosynthesis by the deletion of the *Tac1* gene, (1.35-fold decrease, *p* < 0.01), the pharmacological blockade of NK1R (using L703606, 1.25-fold decrease, *p* < 0.05), or the combination of these two methods (1.30-fold decrease, *p* < 0.01) attenuated the CLP surgery-induced upregulation of the expression of VCAM1 on the vascular endothelial cells in the lungs of mice.

## 3. Discussion

SP plays a significant pro-inflammatory role by promoting leukocyte infiltration in the liver and lungs of mice with CLP surgery-induced sepsis. This study demonstrated for the first time that SP upregulated the expression of both ICAM1 and VCAM1 by activating the NK1R pathway in vascular endothelial cells in the liver and lungs of mice with sepsis induced by CLP surgery. This effect of SP is likely to provide a mechanism to explain how SP promotes leukocyte infiltration, the subsequent inflammatory response, and injury in the liver and lungs during CLP surgery-induced sepsis.

MPO is an enzyme expressed by neutrophils, and its activity has been widely used as a technique for investigating the profile of neutrophil infiltration in various tissues [29]. The activity of MPO in the liver and lungs were measured first to demonstrate the profile of neutrophil accumulation in these tissues of mice. We found that CLP surgery resulted in a significant increase in the activity of MPO in the liver and lungs of mice, in line with previous research [30]. In contrast, suppressing the biosynthesis of SP or its interactions with NK1R attenuated the CLP surgery-induced increased activity of MPO in these tissues of mice, in accordance with previously reported investigations [31,32]. These results suggested that SP played an important role in promoting the recruitment of circulating neutrophils to the liver and lungs of mice with sepsis induced by CLP surgery through priming NK1R.

We then investigated how SP promotes leukocyte infiltration in the liver and lungs of mice with sepsis induced by CLP surgery by focusing on the engagement of the vascular endothelial cells in these tissues. The dysregulation of the liver sinusoidal endothelial cells due to the increased biosynthesis of SP has been shown to make a significant contribution to the pathogenesis of acute liver injury in animals with sepsis [26]. However, the mechanism underlying acute liver injury attributed to SP-induced dysfunction in the liver sinusoidal endothelial cells in sepsis is not completely understood. We found that the increases in the biosynthesis of SP and its interactions with NK1R were associated with the upregulation of ICAM1 and VCAM1 in the liver of mice with CLP surgery-induced sepsis. These results prompted the experiments on the role of SP in the expression of these adhesion molecules on liver sinusoidal endothelial cells of mice with CLP surgery-induced sepsis. In line with previous research [27], the expression of ICAM1 and VCAM1 on the liver sinusoidal endothelial cells in mice with CLP surgery-induced sepsis was significantly upregulated. We also found that the increases in the biosynthesis of SP and its interactions with NK1R were significant contributors to the upregulation of ICAM1 and VCAM1 on vascular endothelial cells in the liver of mice with sepsis induced by CLP surgery. These data point to a key role for SP, by activating NK1R, in the CLP surgery-induced activation of the liver sinusoidal endothelial cells of mice.

Abnormalities in the pulmonary vascular endothelium, including impaired vasodilation and an increased risk of thrombosis, have also been linked to sepsis-associated acute inflammatory injury in the lungs of mice [23,24,25,27]. The increase in the expression of ICAM1 and VCAM1 was shown to exacerbate the inflammatory response and injury in the lungs of mice with sepsis induced by CLP surgery [33]. Similarly, the elevation in the expression of ICAM1 and VCAM1 on pulmonary endothelial cells was identified as a significant contributor to CLP surgery-induced acute inflammatory injury in the lungs of mice [27].

In the present study, the increases in the biosynthesis of SP and its interactions with NK1R were linked to the upregulation of the concentrations of ICAM1 and VCAM1 in the lungs of mice with sepsis induced by CLP surgery. These results led to the investigation of the roles of SP and its interactions with NK1R in the expression of these adhesion molecules on the pulmonary vascular endothelial cells of mice with CLP surgery-induced sepsis. We found that CLP surgery also increased the expression of ICAM1 and VCAM1 on the pulmonary vascular endothelial cells of mice. These results are in agreement with our previous study, in which this increase was shown [27]. Furthermore, we found that the increased biosynthesis of SP and its interactions with NK1R played crucial roles in the upregulation of ICAM1 and VCAM1 on the pulmonary vascular endothelial cells of mice with CLP surgery-induced sepsis. These data showed that SP contributed to the CLP surgery-induced activation of the pulmonary vascular endothelial cells of mice by activating NK1R.

In line with our previous study, treatment with the NK1R antagonist L703606 did not cause any further attenuation of the increases in leukocyte infiltration and the expression of ICAM1 and VCAM1 on the vascular endothelial cells in the liver and lungs in the *Tac1*^−/−^ mice with sepsis induced by CLP surgery [6]. These results suggest that the NK1R-mediated upregulation of the infiltration of leukocytes and the activation of vascular endothelial cells in these tissues of mice with CLP surgery-induced sepsis is mainly driven by SP [6]. Similarly, if the interactions between SP and NK1R were blocked by L703606, the deletion of the *Tac1* gene did not cause any further attenuation of the increases in leukocyte infiltration and the expression of ICAM1 and VCAM1 on the vascular endothelial cells in the liver and lungs of mice with sepsis induced by CLP surgery. These results indicate that SP promotes leukocyte infiltration and activates vascular endothelial cells in the liver and lungs of mice with sepsis induced by CLP surgery primarily by priming NK1R [6].

## 4. Materials and Methods

### 4.1. Group Setting

**Figure 6:**
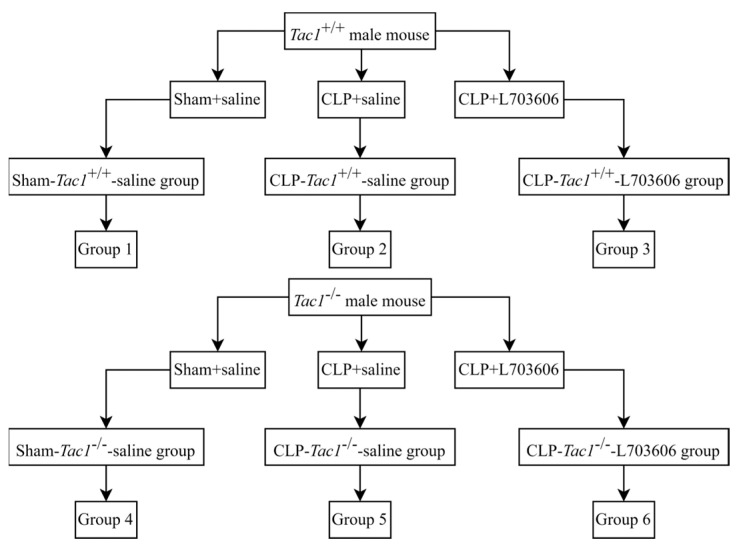
Group setting.

This study is regulated by the Animal Welfare Act and was approved by the University of Otago Animal Ethics Committee (AUP-19-104). As shown in Figure 6, 18 *Tac1*^+/+^ (Balb/c, male) and 18 *Tac1*^−/−^ (Male) mice (8~10 weeks, 20~30 g) were randomly assigned to three groups, which were the sham+saline group, the CLP+saline group, and the CLP+L703606 group, respectively (*n* = 6 each). *Tac1*^+/+^ mice were purchased from the Christchurch Animal Research Area (CARA). *Tac1*^−/−^ mice were a gift from Prof. AI Basbaum (University of California, San Francisco, CA, USA) and bred as described previously [6,34].

### 4.2. Sepsis Establishment, Manipulations, and Sample Collection from Mice

Sepsis was established in mice by CLP surgery using a previously reported protocol [6]. L703606, a highly specific and potent antagonist of NK1R [35], was dissolved in DMSO and then diluted using sterile saline before experiments. Then, 0.2 mL of saline or L703606 (4 mg/kg) was administered by peritoneal injection to all mice 30 min before the sham or CLP surgery [6]. Before surgery, mice were anesthetized by inhaling 2% isoflurane. During the operation, inhaled isoflurane was reduced to 1%. Aseptic surgical techniques were applied during surgery. Firstly, the abdominal area was disinfected after the abdominal fur was shaved. Secondly, the caecum was exposed by making a small midline incision through the skin and peritoneum of the abdomen. Thirdly, the cecum was ligated with silkam 5.0 at the designated position (1 cm from the tip of the cecum) without occluding the bowel passage. The cecum was then perforated at the distal end using a 22-gauge needle to make a through-and-through puncture. Subsequently, a small amount of stool was squeezed out through both holes. Thereafter, the bowel contents were repositioned in the abdomen and the wound was sutured using permilene 5.0 thread. Mice in the sham operation group underwent the same operation without the CLP procedure. Saline was subcutaneously injected into all mice at the end of the operation. Buprenorphine (Temgesic, 0.2 mg/kg) was given to all mice 45 min before and 3 h after surgery for analgesia by subcutaneous injection. Eight hours after the sham or CLP surgery, mice were euthanized by intraperitoneally injecting a lethal dose of sodium pentobarbital (150 mg/kg). The liver and lung tissues were either fixed with 10% neutral phosphate-buffered formalin or frozen in liquid nitrogen and then stored at −80 °C in the freezer.

### 4.3. Measurement of the Activity of MPO in Tissues

The activity of MPO in the liver and lungs was measured according to a previously reported protocol with minor modifications [27]. Briefly, tissue samples were thawed, snipped, and homogenized in 20 mM phosphate buffer (pH 7.4). Subsequently, tissue homogenates were centrifuged at 4 °C for 10 min (13,000× *g*), and the resulting pellet was resuspended in 50 mM phosphate buffer (pH 6.0). These suspensions were sonicated for 40 s on ice, followed by being subjected to three freezing and thawing cycles, and spun at 4 °C for 10 min (20,000× *g*). The reaction mixture was prepared by mixing 50 µL of the supernatant and 100 µL of freshly prepared 3,3′,5,5′-tetramethylbenzidine solution. These reaction mixtures were incubated at room temperature for 10 min and the reaction was terminated by adding 50 µL of 2 N sulfuric acid. Absorbance was measured at 450 nm and 570 nm (for correction) using a microplate spectrophotometer (Thermo Fisher Scientific, Waltham, MA, USA). The corrected absorbance was further normalized to the protein content of these tissues measured by the Braford protein assay. Results were presented as fold increases over the sham-operated controls.

### 4.4. Measurement of the Concentrations of ICAM1 and VCAM1 in Tissues

The concentrations of ICAM1 and VCAM1 in the liver and lungs of mice were measured using the sandwich ELISA method by following the instructions of the DuoSet ELISA kits (R&D System, Minneapolis, MN, USA). The concentrations of ICAM1 and VCAM1 in the liver and lungs were corrected for the total protein content of these tissues. Results were presented as pg or ng per mg protein.

### 4.5. Measurement of the Expression of ICAM1 and VCAM1 on Vascular Endothelial Cells in Tissues by Double Immunofluorescence Staining

Liver sinusoidal endothelial cells were visualized by staining with LYVE1 (green fluorescence) [36]. Pulmonary endothelial cells were visualized by staining with CD31 (green fluorescence) [37]. Formalin-fixed liver and lung tissues were processed using a tissue processor (Leica Biosystems, Wetzlar, Germany) and embedded in paraffin wax using an embedding station (Leica Biosystems, Wetzlar, Germany). Tissue sections (4 µm) were prepared using a microtome (Leica Biosystems, Wetzlar, Germany), followed by mounting onto adhesive microscope slides (Trajan Scientific and Medical, Ringwood, Australia). Then, these tissue sections underwent a process of deparaffinization and rehydration. These pre-processed tissue sections underwent an antigen retrieval process completed in a pressure cooker (100 °C, 4 min) with Tris-EDTA buffer (pH 9.0), followed by cooling down to room temperature in the buffer (~2 h). These sections were then permeabilizated by PBS with 0.25% Triton (*w*/*v*) for 15 min at 4 °C. After washing with PBST, tissue sections were blocked with 5% bovine serum albumin in the permeabilization buffer for one hour at room temperature. Subsequently, these tissue sections were incubated with appropriate dilutions of the primary antibodies (Table 1) overnight at 4 °C in a humidified chamber. After washing with PBST, these sections were incubated with appropriate dilutions of a FITC-conjugated secondary antibody and Texas red-conjugated secondary antibody (Table 1) with 2 µg/mL 4′,6-diamidino-2-phenylindole (blue fluorescence, nuclear) for one hour at room temperature in the humidified chamber in the dark. After washing with PBST, ~40 L prolong glass antifade mountant was added to each slide and covered with a coverslip. Confocal images were taken using a confocal microscope (Carl Zeiss, Aalen, Germany). The mean fluorescence intensity of ICAM1 (red fluorescence) and VCAM1 (red fluorescence) on the vascular endothelial cells in the liver and lungs was determined by an automated region of interest selection method based on signal threshold criteria using ImageJ software (version 1.54d) by following a previously reported protocol with minor modifications [38].

### 4.6. Statistical Analysis

Data are presented as means ± SDs or means ± SEMs. All the statistical analyses in this investigation were performed using the GraphPad Prism software (version 9.2.0, GraphPad Software Incorporated, San Diego, CA, USA). All data were analysed for normal distributions by the Shapiro–Wilk test. One-way or two-way analysis of variance with post hoc Tukey’s multiple comparisons test were conducted with the normally distributed data to compare multiple groups. Statistical significance was set as a *p*-value < 0.05.

## 5. Conclusions

**Figure 7:**
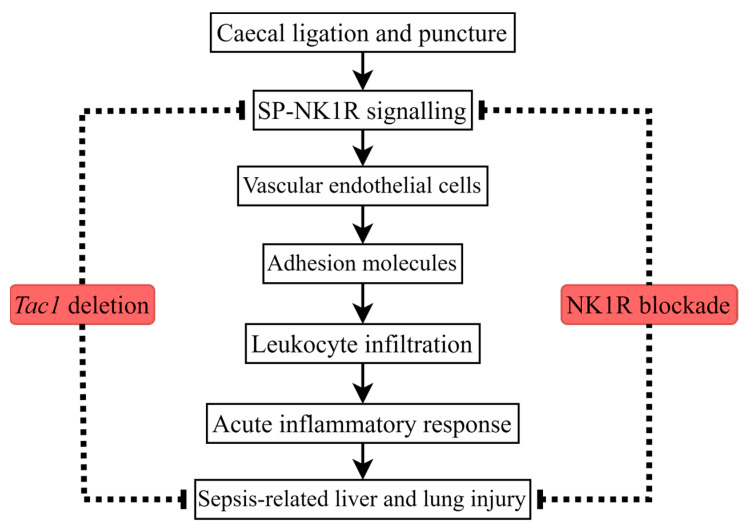
CLP surgery promotes leukocyte infiltration, leading to an acute inflammatory response and injury in the liver and lungs of mice. The pro-inflammatory actions of SP in mice with sepsis induced by CLP surgery are mainly mediated by NK1R. The impact of SP on upregulating the expression of adhesion molecules on vascular endothelial cells in the liver and lungs is likely associated with the role of SP in increasing leukocyte infiltration in theses tissues of mice with sepsis induced by CLP surgery.

This study demonstrated that the upregulation of the expression of ICAM1 and VCAM1 on vascular endothelial cells in the liver and lungs is associated with the SP-induced increase in leukocyte infiltration in these tissues of mice with CLP surgery-induced sepsis (Figure 7). This study supports the hypothesis that, during CLP surgery-induced sepsis, SP is the main ligand responsible for NK1R activation, which, in turn, is the primary receptor mediating the effects of SP [6]. Collectively, attenuating the increased infiltration of leukocytes in the liver and lungs by attenuating the SP-induced upregulated expression of ICAM1 and VCAM1 on vascular endothelial cells in these tissues could be developed as a novel adjuvant treatment for sepsis-associated liver and lung injury.

## Figures and Tables

**Table 1:** Information on the antibodies used in this study.

Antibody	Dilution	Source, Catalogue No.
ICAM1	1:1000	R&D Systems, Minneapolis, MN, USA
VCAM1	1:200	R&D Systems, Minneapolis, MN, USA
LYVE1	1:200	Abcam, Cambridge, UK
CD31	1:100	Abcam, Cambridge, UK
FITC-conjugated secondary antibody	1:500	Abcam, Cambridge, UK
Texas red-conjugated secondary antibody	1:500	Abcam, Cambridge, UK

## Data Availability

All data generated or analysed during this study are included in this published article.
